# Outcome of Root Canal Treatments Using a New Calcium Silicate Root Canal Sealer: A Non-Randomized Clinical Trial

**DOI:** 10.3390/jcm9030782

**Published:** 2020-03-13

**Authors:** Angelo Zavattini, Alan Knight, Federico Foschi, Francesco Mannocci

**Affiliations:** 1 Department of Endodontics, Faculty of Dentistry, Oral & Craniofacial Sciences. Floor 22 Tower Wing, Guy’s Dental Hospital, London SE1 9RT, UK; eknight@hotmail.co.uk (A.K.); federico.foschi@kcl.ac.uk (F.F.); francesco.mannocci@kcl.ac.uk (F.M.); 2 Department of Therapeutic Dentistry I. M. Sechenov First Moscow State Medical University, 119146 Moscow, Russia

**Keywords:** root canal, biosilicate cement, bioroot, single cone, CBCT, warm vertical condensation, hydraulic cements

## Abstract

Background: The aim of this study was to compare the success rate of root canal treatments undertaken using a calcium silicate root canal sealer in combination with a single cone with non-calcium silicate cement and warm vertical condensation. Methods: 150 necrotic or pulpitic teeth were treated. (REC: 08/H0804/79). Following standardized root canal chemo-debridement. The canals were obturated using warm vertical condensation of gutta-percha and epoxy-based sealer (AH plus) or a calcium silicate sealer (BioRoot^TM^ RCS) with a single cone technique. Follow-up assessment was conducted at 12 months using Cone Beam Computed Tomography (CBCT). Results: At 1-year recall, 104 teeth were assessed (51 AH plus, 53 BioRoot^TM^ RCS). The success rate using loose criteria for the CBCT images and PA radiographs was respectively 80% and 89% in the AH plus/warm vertical condensation group, 84% and 90% in the BioRoot^TM^ RCS/single cone group. There was no statistically significant difference between the two groups (Fisher exact test *p* value 0.6099 for the CBCT images). Conclusion: Within the limitations of this non-randomized trial, a calcium silicate cement in combination with single cone resulted in a similar proportion of successful cases compared to warm vertical condensation and epoxy-based sealer.

## 1. Introduction

The obturation of a thoroughly cleaned and shaped root canal system is the ultimate clinical objective of the endodontic therapy besides an effective disinfection. It requires sound knowledge and technical skills and its quality can affect the outcome of the procedure [1].

The three-dimensional obturation of the root canal system is important to entomb residual bacteria and preventing any bacterial re-contamination that would lead to relapse of the apical periodontitis [2].

Root canal obturation is routinely accomplished with the use of either “cold” or “warm” gutta-percha condensation techniques in conjunction with a sealer which act as lubricant, help to seal off voids and potentially seal any accessory canal [3].

Single cone obturation technique has been often regarded as inadequate due to the potential for apical leakage [4], however with the recent advent of hydraulic cement-based endodontic sealer their acceptance has increased because of their increased properties [5]. The possibility of a dissolving process in the apical third may contribute to re-growth of microorganisms and reinfection, however it appears that the solubility of a calcium silicate-based sealer is linked to a positive biological outcome.

In the single cone technique, the root canal is generally obturated with a fitted cone that best matches the shape (taper and apical gauge) of the last rotary instrument used in combination with large quantity of sealer [6].

The potential for cementoblasts bio-mineralization and bacteriostatic action of calcium silicate sealers may allow an increased and prompter healing of endodontic infections [7,8].

Calcium silicates-based sealers have been found to be able to promote apical healing, to possess antibacterial activity, and to bond to tooth structure. Their biological properties depend on their chemical composition and their setting reaction which consist of a hydration reaction followed by a precipitation reaction of calcium phosphate and formation of hydroxyapatite [9].

At the present time, however, the success rate of root canal obturation carried out using calcium silicate or non-calcium silicate sealers has not yet been compared in a clinical trial.

The success rate of root canal treatments is normally assessed using clinical and radiographic parameters. The radiographic success is identified by the prevention of the development, reduction in size, or complete disappearance of apical radiolucency, indicative of apical periodontitis [10].

Cone beam computed tomography (CBCT) was used to assess the success of root canal treatments in recent clinical trials [11,12,13] and it was found to have higher sensitivity and specificity than conventional periapical radiography in the detection of apical radiolucency, as confirmed in representation of the actual periapical histological status, as per a study carried out in fresh cadavers [14].

This non-randomized controlled clinical trial compared the success rate of root canal obturations undertaken using a calcium silicate root canal sealer BioRoot^TM^ RCS (Septodont, Saint Maur Des Fosses, France) in combination with a single cone technique with that of obturations undertaken with an epoxy resin-based sealer AH Plus (Dentsply DeTrey, Konstanz, Germany) used in combination with a warm vertical condensation of gutta-percha.

The null hypothesis was the following: there is no difference in the success rate of root canal treatments carried out with a single cone technique and a calcium silicate sealer (BioRoot^TM^ RCS) or a warm vertical condensation technique and a resin epoxy sealer (AH plus).

## 2. Materials and Methods

The study was conducted in compliance with the principles of the Declaration of Helsinki and Good Clinical Practice after approval from the London Westminster research ethics committee (REC: 08/H0804/79). Patient information sheets were distributed, and informed written consent obtained prior to study enrolment.

Restorable teeth diagnosed with irreversible pulpitis or necrotic pulp were selected. Teeth with signs of apical resorption that would have rendered rotary files clinically inappropriate for use were excluded. In addition, roots with open apices (immature teeth), blocked canals, iatrogenic perforations, fractures, and those apices which were over-enlarged at the time of pulp extirpation together with those that were deemed clinically inappropriate to be shaped rotary file were excluded.

A total of 150 teeth fulfilled the inclusion criteria. Twenty-five teeth were subsequently excluded as consent was withdrawn (2 teeth) or because upon commencing treatment, tooth was deemed unrestorable (23 teeth).

Root canal treatments were performed by specialists or senior specialist trainees under the direct supervision of specialist endodontic staff at Guy’s and St Thomas’ NHS Foundation Trust, London, UK. All operators were provided with a standardized step-by-step protocol sheet. 

Details of the clinical diagnosis and radiographic assessment of recalled teeth are present in Table 1 and Table 2.

A CBCT scan and long cone periapical (LCPA) radiographs were both available to be assessed prior to and during root canal retreatment. The LCPA were taken using a beam aiming device X-ray unit (Heliodent, Sirona, Benshein Germany) with paralleling technique at exposure parameters of 65 kV and 7 mA and photostimulable phosphor plates (Digora Optime; Soredex, Tuusula Finland). CBCT scans were taken of the area of interest (3D Accuitomo 80; J Morita, Kyoto, Japan). The exposure parameters were 90 kV, 3–5 mA, exposure time 17.5 s, voxel size 0.08 mm, and a small field of view (FOV) of 40 mm × 40 mm was employed to limit radiation exposure. All scans were reformatted (0.125 slice intervals and 1.5 mm slice thickness). A dental operating microscope was used during treatments.

All primary root canal treatments were performed using sterilized, single use, endodontic files. A standardized protocol was used to disinfect and fill the root canal system. Each canal was initially negotiated with size 08 and 10 stainless steel Flexofiles (Dentsply Maillefer, Ballaigues, Switzerland). 

A balanced force instrumentation technique was used to negotiate each canal to its provisional working length. The definitive working lengths were determined with the aid of an apex locator (Root ZX II; J Morita, Kyoto, Japan) in conjunction with measurements using the CBCT software (I-Dixel; J Morita). 

The working lengths were always 1 mm short of the “0” apex locator reading length. Canals were then prepared to at least a size 20 Flexofile to the working length, after which ProTaper Next nickel-titanium rotary instruments (Dentsply Maillefer) at 300 RPM were used in a crown-down approach to prepare each root canal to at least a X2 master apical rotary file. 

Canals were irrigated throughout the instrumentation with 2% sodium hypochlorite (Chloraxid 2.0%; PPH Cerkamed, Sandomierska, Poland) for 30 min, the irrigants were replenished every 3–4 min after which they were immediately agitated with an appropriately selected gutta-percha point extending to 2 mm short of the working length for approximately 30 s. The root canals were then irrigated with 15% ethylenediaminetetraacetic acid (EDTA) (ENDO-Solution; PPH Cerkamed) followed by a final irrigation with sodium hypochlorite.

The irrigants were ultrasonically energized with a size 25 Endo-Activator (Dentsply Maillefer) for 1 min. 

The canals were then dried with paper points and the operators following an alternate sequence, obturated the canals using warm vertical condensation of gutta-percha and AH Plus Root Canal Sealer (Dentsply DeTrey, Konstanz, Germany, 63 teeth) or with a single cone technique and BioRoot^TM^ RCS (Septodont, Saint Maur Des Fosses, France, 62 teeth).

The teeth were then restored with permanent composite resin (Herculite Ultra; Kerr Corporation, Orange, CA, USA). All teeth requiring permanent, full cuspal-coverage were restored by the referring practitioner within 1 month of completion of the root canal treatment.

### Follow-Up Assessment

All patients were contacted approximately 11 months post treatment to schedule a 12-month review appointment. Clinical assessment included patients reported symptoms, tenderness to percussion test; mobility and periodontal probing depths were determined. The soft tissues were also assessed for tenderness to palpation, signs of erythema, and presence of fistulous tracts; the integrity and marginal fit of the definitive restoration were also assessed.

A consensus panel of 2 trained, calibrated, experienced endodontists assessed the CBCT radiographs. 

The reliability of the consensus panel was evaluated by jointly repeating the assessment of radiographic images after 4 w. The inter-examiner agreement was evaluated by individual randomized assessment of 50% of the LCPA/CBCT images and repeated after 4 w.

Each root was examined for the presence, absence, and change (increase/decrease) in size of any periapical radiolucency (PA). A periapical radiolucency was defined as exceeding the widening of the periodontal ligament (PDL) space. Widening of the PDL space was defined as less than double that of the equivalent healthy PDL space of the adjacent healthy teeth. A *PA* lesion was defined as radiolucency associated with the radiographic apex of the root, larger twice as much the width of the healthy PDL space. 

Teeth were classified into 2 outcomes categories as follow:

1. Successful: functional and asymptomatic teeth with absence or decreased size of PA radiolucency.
2. Failed: non-functional, symptomatic teeth with unchanged, new, or enlarged PA radiolucency.

A Fisher’s Exact test was used to determine the difference between the number of successful and unsuccessful outcomes in the two groups. The significance level was set at α = 0.05. 

The sample size was determined by assessing previous similar research. It was calculated that 150 teeth would provide 80% power to show a 25% difference in the number of lesions identified as present between the radiological systems.

**Figure 1:**
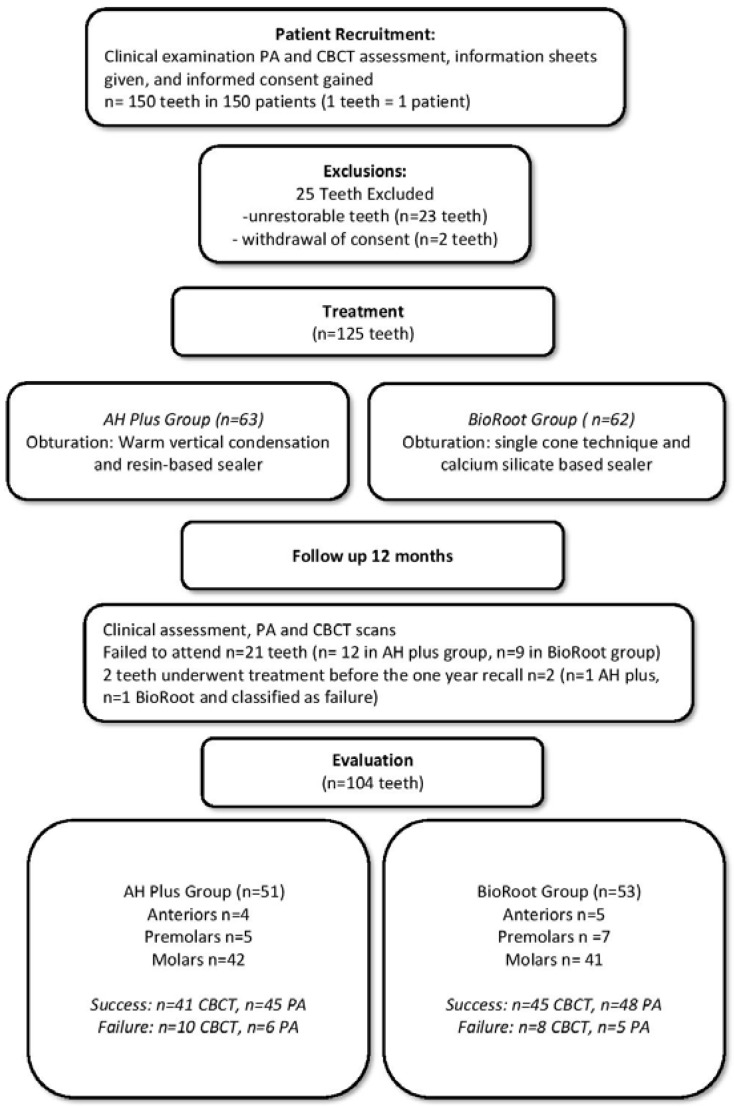
Flow diagram of the study recruitment, follow-up, and results.

A flow diagram of recruitment and follow-up of participants is shown in Figure 1.

## 3. Results

A total of 150 patients (150 teeth) were recruited following the exclusions described in the flowchart (Figure 1). Twenty-five teeth were subsequently excluded because consent was withdrawn (2 teeth) or because upon commencing treatment, the tooth was deemed unrestorable (23 teeth). A total of 125 teeth underwent complete root canal treatment (63 AH plus/62 BioRoot^TM^ RCS plus). 

At the one-year recall, 21 teeth failed to attend for review (12 AH plus/9 BioRoot^TM^ RCS) and 2 teeth underwent further re-treatment before the one-year recall (1bioroot 1 AH plus), leaving a total of 104 for the final assessment (including the 2 that had been re-treated before the recall date which were classified as failures). 

For the AH plus arm, a total of 51 teeth were assessed (5 premolars, 4 anteriors, 42 molars), whereas 53 teeth were assessed in the BioRoot RCS arm (7 premolars, 5 anteriors, 41 molars). 

The success rate using loose criteria for the CBCT images was 80% in the AH plus/warm vertical condensation group (41 success/10 failure) and 84% in the BioRoot^TM^ RCS /single cone group (45 success 8 failure). 

The success rate using loose criteria for the periapical radiographs was 89% in the AH plus/warm vertical condensation group (45 success/6 failure) and 90%in the BioRoot^TM^ RCS/single cone group (48 success/5 failure). 

The difference between the two groups was found to be not statistically significant (Fisher exact test *p* value 0.6099 for the CBCT images and 0.7582 for the periapical radiographs). 

No adverse event was reported during this clinical trial. 

## 4. Discussion

**Figure 2:**
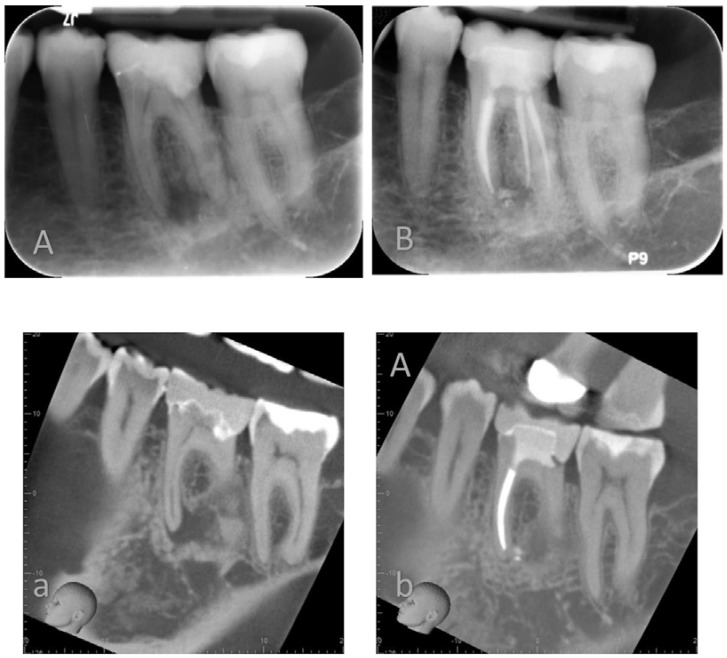
Lower left mandibular first molar root filled using a warm vertical condensation technique and AH plus root canal sealer. Preoperative Periapical X-ray (**A**) and Cone Beam Computer Tomography (**a**) and recall PA X-ray (**B**) and CBCT (**b**) at 12 months.

**Figure 3:**
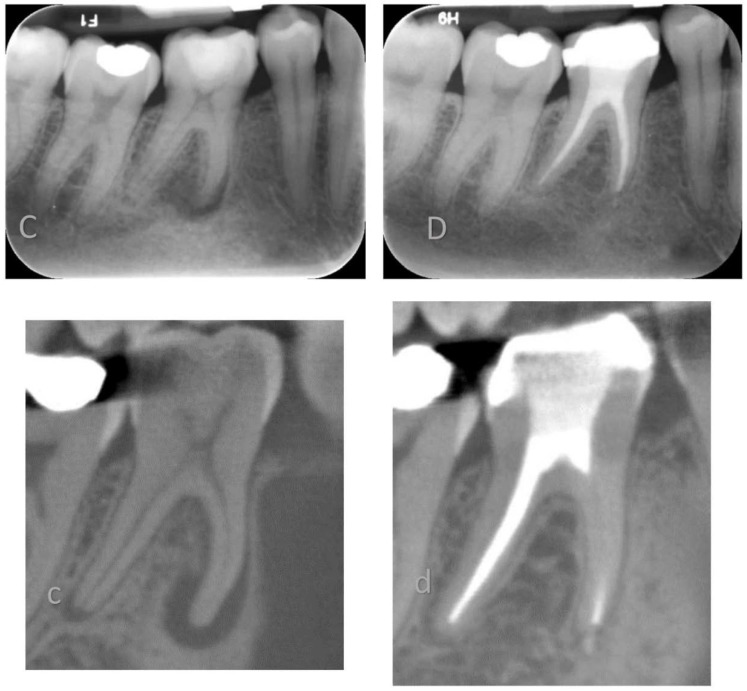
Lower right mandibular first molar root filled using a single cone technique and BioRoot^TM^ RCS. Preoperative PA X-ray (**C**) and CBCT (**c**) and recall PA X-ray (**D**) and CBCT (**d**) at 12 months.

In this non-randomized clinical trial, the clinical outcome of two different root canal obturation techniques was compared with CBCT imaging at one-year follow (Figure 2 and Figure 3). The patient population was enrolled and treated in a teaching hospital by senior postgraduate trainees under specialists’ supervision.

Several previous clinical studies determined the endodontic outcome following root canal treatment with cold lateral and warm condensation techniques, relying on LCPA assessment [15,16]. The limited sensitivity of 2D intraoral radiography may have led to type II errors in assessing significant differences due to the use of alternative obturation techniques (i.e., cold lateral vs. warm vertical condensation technique). For this reason, it is still debated which is to be considered the gold standard in terms of root canal obturation technique. The current trends in endodontic teaching, especially at undergraduate level, are shifting from the cold lateral condensation technique to single cone obturation technique due to practicality consideration. A recent study revealed diminished chances of procedural errors when using single cone obturation technique in conjunction with BioRoot^TM^ RCS [17]. This epochal change in obturation approaches has not been substantiated by any high-level hierarchy of evidence study, in particular no clinical study has been carried out to compare the outcome of root canal treatment carried out with warm vertical condensation technique compared to single cone obturation used in conjunction with hydraulic cements. 

The favorable characteristics of hydraulic cements, often mislabeled as bioceramic, used as endodontic sealer presents with several bioactive properties, including potential for hydroxyapatite formation, mineralization of dentinal structure, alkaline pH, and sealing properties. These characteristics may allow clinical use in a single cone technique [18]. 

The advent of NiTi rotary instruments allowed, for predictable taper and apical gauge preparation, which can be matched by industrially produced gutta-percha cones that fit the prepared canal with an accurate dimensional tolerance. The use of traditional sealer in conjunction with single cone technique has been discouraged due to several in vitro studies showing high bacterial percolation rate and increased dye leakage. The validity of in vitro microbial percolation and dye leakage studies has been criticized due to potential procedural errors [19,20]. Current evidence is lacking with modern hydraulic cement. However, several studies proved the high hermetic seal of calcium silicate cements, encouraging their use to create apical plugs as sole obturation material or in conjunction with gutta-percha [6,21].

A successful outcome in endodontics is multifactorial depending on the completion of several stages during the root canal treatment [15]. The use of an effective irrigation protocol with adjunct activation regimes is essential to achieve a good outcome. [22] The eradication of the endodontic biofilm is followed by the creation of a hermetic root canal filling. Subsequently, the successful provision of a definitive restoration with adequate coronal seal is of paramount importance. In the present study, the chemo-debridement protocols followed the current best practice, including irrigant activation. The provision of a final restoration involving a cuspal coverage, when required, was carried out in due time, within a month in case of indirect restorations.

Within the limitations of this non-randomized clinical trial, BioRoot^TM^ RCS in combination with a single cone technique resulted in a similar proportion of successful cases compared to warm vertical condensation and AH plus.

The results of our study are comparable to those of previous studies evaluating the outcome of non-surgical endodontic treatments using periapical radiographs [15,16] and CBCT [12] for the assessment of the outcome of endodontic treatments. 

In addition, CBCT was used to assess the clinical outcome, representing an advantage in terms of accuracy when evaluating the outcome of root canal treatment in comparison with 2D radiographic approaches. On this note, only one cohort study evaluated the outcome of non-surgical root canal treatments using a single cone technique and a tricalcium silicate-based sealer [23]. The authors retrospectively evaluated 307 teeth using PA radiograph over a period of 6 years and reported an overall success rate of 90%. 

Our findings are in line with the range of results reported in the literature, suggesting that BioRoot^TM^ RCS in combination with a single cone technique can achieve a predictable success rate in the range of 84% to 90% at one-year of follow-up.

To the authors’ knowledge this is the first clinical trial comparing the outcome on non-surgical endodontic treatments using tricalcium silicate endodontic sealer and single cone technique with warm vertical condensation of gutta-percha and sealer.

The non-randomized nature of the trial may limit the external validity of this study; however, the two groups were similar in terms of tooth type treated and experience of the operators. In addition, all root canal treatments were undertaken in 2 visits and the study was conducted in an educational center where a postgraduate student worked under supervision and followed the same instrumentation and irrigation protocol thereby minimizing differences between operators.

## 5. Conclusion

Within the limitations of this non-randomized trial, BioRoot^TM^ RCS (Septodont) in combination with single cone resulted in a comparable success rate of cases compared to that of warm vertical condensation and AH plus (Dentsply DeTrey, Konstanz).

## Figures and Tables

**Table 1:** Clinical diagnosis of recalled teeth (*n* = 125) at baseline.

Group	Diagnosis
**AH Plus**	42 necrotic
	9 Irreversible pulpitis
**BioRoot^TM^**	47 Necrotic
	6 Irreversible Pulpitis

**Table 2:** Radiographic assessment (presence of lesions) of recalled teeth (*n* = 125) at baseline.

Group	LCPA	CBCT
**AH plus (*n* = 51)**	43 lesions present	48 lesions present
	8 lesions absent	3 lesions absent
**BioRoot^TM^ (*n* = 53)**	41 lesions present	46 lesions present
	12 lesions absent	7 lesions absent

LCPA, long cone periapical; CBCT, cone beam computed tomography.
